# Genome Sequence of a Novel Archaeal Fusellovirus Assembled from the Metagenome of a Mexican Hot Spring

**DOI:** 10.1128/genomeA.00164-13

**Published:** 2013-04-25

**Authors:** Luis E. Servín-Garcidueñas, Xu Peng, Roger A. Garrett, Esperanza Martínez-Romero

**Affiliations:** Department of Ecological Genomics, Center for Genomic Sciences, National University of Mexico, Cuernavaca, Morelos, Mexicoa;; Department of Biology, Archaea Centre, University of Copenhagen, Copenhagen, Denmarkb

## Abstract

The consensus genome sequence of a new member of the family *Fuselloviridae* designated as SMF1 (*Sulfolobales* Mexican fusellovirus 1) is presented. The complete circular genome was recovered from a metagenomic study of a Mexican hot spring. SMF1 exhibits an exceptional coding strand bias and a reduced set of fuselloviral core genes.

## GENOME ANNOUNCEMENT

Members of the *Fuselloviridae* family from the crenarchaeal order *Sulfolobales* have been characterized, and they are abundant in extreme geothermal environments (1, 2). They carry circular double-stranded DNA (dsDNA) genomes and exhibit spindle-shaped morphologies. Here, we report the consensus genome sequence of a novel fusellovirus recovered from aqueous sediments from Los Azufres, Mexico.

Samples were collected from a hot spring with a pH of 3.6 and a temperature of 65°C. DNA was purified using the UltraClean microbial and the UltraClean Mega soil DNA kits (MoBio Laboratories, Inc., Carlsbad, CA). Sequencing was performed on an Illumina GAIIx platform, producing 36-bp paired-end reads with 300-bp inserts representing 216 Mb. Reads were assembled using Velvet 1.2.07 (3). A set of contigs were predicted by BLASTX searches to be of fuselloviral origin. Gaps were closed iteratively by mapping and reassembling reads to these contigs using Maq 0.7.1 (4) and Velvet. Open reading frames (ORFs) were predicted using GeneMark.hmm2.0 (5) and were manually verified using Artemis (6).

The average sequence coverage of the 14,847-bp circular dsDNA genome was 1,257-fold. We detected 57 candidate single nucleotide polymorphisms by Maq. The G+C content was 45.43%, higher than the 37.5 to 39.7% content of other fuselloviral genomes (1, 2, 7–9).

The genome has a strong coding-strand bias, not previously seen for fuselloviruses, with only the ubiquitous fuselloviral integrase encoded on one strand. The gene organization is also exceptional for fuselloviruses, with a high incidence of genes arranged in operons, which are also likely to encode cofunctional proteins.

Twenty-four genes were predicted, 22 of which are arranged in five operons. Fourteen genes have putative fuselloviral homologs, consistent with SMF1 being a member of the *Fuselloviridae* family. Most gene products show 30 to 70% amino acid sequence similarity to the best fuselloviral matches. Previous studies identified thirteen genes conserved in all fusellovirus genomes (2), and nine of these were localized in a “core” genomic region of SMF1. The core genes encode a DnaA-like protein, the integrase, one VP1-like structural protein, a putative helix-turn-helix (HTH) transcriptional regulator, and five proteins with unknown functions.

Five additional putative gene products shared with other fuselloviruses include a second VP1-like protein, a VP2-like structural protein, a putative end-filament protein, a regulatory protein, and a hypothetical protein. Three further nonconserved ORF products showed sequence similarities to putative regulatory proteins.

The host of SMF1 is likely to be a member of the order *Sulfolobales*. Fuselloviruses can replicate in both *Sulfolobus* and *Acidianus* species of the order *Sulfolobales* (2), and they are predicted to have an extended host range that may include as-yet-uncultured species (10).

In conclusion, the SMF1 genome was recovered from a site widely separated geographically from the locations of other sequenced fuselloviruses. The SMF1 genome shows exceptional properties, including a coding-strand bias and a high incidence of genes organized in operon structures, but nevertheless, it retains a large set of conserved fusellovirus genes, which lends further support to the exchange of genetic material over intercontinental distances (2, 10).

### Nucleotide sequence accession number. 

The genome sequence was deposited in GenBank under the accession no. KC618393.
